# Mental disorders at the beginning of adolescence: Prevalence estimates in a sample aged 11-14 years

**DOI:** 10.1016/j.puhip.2022.100348

**Published:** 2022-12-02

**Authors:** Christin Scheiner, Jan Grashoff, Nikolaus Kleindienst, Arne Buerger

**Affiliations:** a Department of Child and Adolescent Psychiatry, Psychosomatics and Psychotherapy University Hospital of Würzburg, Germany; b German Center of Prevention Research in Mental Health, Germany; c Faculty of Psychology and Neuroscience, Maastricht University, Netherlands; d Central Institute of Mental Health, Department of Psychosomatic Medicine and Psychotherapy, Medical Faculty Mannheim, Heidelberg University, Mannheim, Germany

**Keywords:** Adolescence, Mental health, Prevalence, NSSI, Depression, Eating disorders, STBs

## Abstract

**Objectives:**

This study aims to provide a deeper insight into mental disorders in early adolescence. We report prevalence rates (mental health problems, depressive symptoms, eating disorders, NSSI, STBs) to be used in future studies and clinical ventures. We also expected to find gender differences, with girls being be more affected than boys are.

**Study design:**

877 adolescents (M = 12.43, SD = 0.65) from seven German high schools completed a series of questionnaires assessing their mental health (SDQ, PHQ-9, SEED, DSHI-9, Paykel Suicide Scale, FAS III).

**Methods:**

We calculated cut-off-based prevalence estimates for mental health issues for the whole sample and compared estimates between genders.

**Results:**

12.5% of the sample reported general mental health problems. The estimated prevalence of depressive symptoms lay at of 11.5%. Additionally, 12.1% and 1.3% of the participants displayed relevant symptoms of anorexia or bulimia nervosa, respectively. A total of 10.8% reported engaging in non-suicidal self-injury (NSSI) at least once in their lifetime, of whom 5.6% reported repetitive NSSI. 30.1% of the participants described suicidal thoughts, 9.9% suicide plans, and 3.5% at least one suicide attempt. Girls were generally more affected than boys, except for bulimia nervosa, suicidal behavior, and partly NSSI.

**Conclusion:**

Our findings corroborate the established relevance of early adolescence for the development of mental health problems and suggest that a substantial proportion of young adolescents suffer from such problems early on. Considering the ongoing COVID-19 pandemic and reported negative mental health consequences, the current findings underline the importance of preventive interventions to avoid the manifestation of mental disorders during adolescence.

## 1 Introduction

Early adolescence (11-14 years) marks a crucial period for the development and onset of mental health problems, which often manifest as disorders in the course of adulthood if they are not dealt with early on [1]. An international meta-analysis involving 27 countries found a pooled prevalence of 13.4% for mental disorders among children and adolescents [2], corroborated by estimates of around 20% reported by the Word Health Organization [3]. Recent epidemiological data show that the proportion of individuals with onset of any mental disorders before the age of 14 lies at 34.6%, which rises to 48.4% up to the age of 18 [1]. Various gender differences regarding the onset and type of mental disorders can be observed, especially in childhood and adolescence [[4], [5], [6]], potentially due to differing vulnerabilities depending on gender [7]. The high reported prevalence estimates and the early onset of mental disorders before the age of 14 years emphasize the importance of investigating the underlying processes and associations in this particularly vulnerable group. This is in line with a statement made by the WHO: Good mental health is accordingly defined as “a state of well-being in which the individual is able to realize their own capacities, cope with the normal pressures of life, work productively and fruitfully, and make a contribution to his or her community” [8,9]. This means that not having a mental disorder is not equivalent to being mental healthy and research suggests that the use of preventive measures is maximized when one intervenes at the time of the onset of mental disorders [1]. Hence, we need to find out more about the onset of mental health issues and the gender differences in order to establish well-chosen prevention programs at the right time.

The aforementioned gender differences appear to vary according to age. For instance, a German study reported that mental disorders were diagnosed earlier in boys, and mainly consisted of externalizing disorders such as attention deficit hyperactivity disorder (ADHD) or developmental disorders [10]. With the onset of adolescence, there appears to be a measurable shift, with more internalizing disorders such as depression and anxiety disorders being diagnosed, predominantly in girls [10,11] and with an increased incidence at around the age of 14 [12]. Latent growth curve modeling of depressive symptoms indicated that girls’ symptoms accelerated early in adolescence whereas boys’ symptoms accelerated later (with respect to internalizing disorders), with more girls (24%) experiencing an episode of major depression or dysthymia by the age of 20 [13]. Yet, these dynamics are not well explained or understood.

Taking a closer look at specific disorders such as depression, the prevalence in adolescents increased from 8.7% in 2005 to 11.3% in 2014 [14]. The incidence of depression increases rapidly during adolescence [15], with gender differences in major depression appearing by the age of 12 (OR 2.37) and peaking at ages 13–15 (OR 3.02) [16]. A recent meta-analysis confirmed these substantial gender differences, reporting that depression was more common in girls than in boys (2.41% vs. 0.92%), with a peak at around 14 years [17]. In addition, depression is associated with difficulties across the lifespan, such as failure to complete secondary school (OR 1.76) or unemployment (OR 1.66) [18].

Another type of mental disorders with onset in early adolescence is eating disorders such as anorexia or bulimia nervosa (AN and BN; [19]). In a cross-sectional survey in German schools, the authors reported a 12-month prevalence of 0.3% for full-syndrome AN, 10.9% for partial-syndrome AN, 0.4% for full-syndrome BN and 0.2% for partial-syndrome BN [20]. However, prevalence rates for body image symptoms such as fear of weight gain or overvaluation of body weight are estimated to be much higher in adolescents (14.3-25.7%) [20]. Furthermore, AN and BN tend to become chronic, can negatively influence socioeconomic achievement, and are often associated with serious physical complications as well as high rates of morbidity and mortality [21,22]. In individuals aged under 15 years, both AN and BN are substantially more frequent in girls than in boys, with sex ratios of 5 to 0 (AN) and 5 to 1 (BN) [20]. These findings suggest body and weight concerns to be especially important in female adolescents, warranting an updated investigation of these issues.

Transdiagnostically, there are other high-risk behaviors in adolescence that increase the likelihood of developing mental disorders, such as dysfunctional coping strategies [23]. Maladaptive emotion regulation strategies like rumination, alcohol or drug use, and non-suicidal self-injury (NSSI) are often associated with depression or even eating disorders [24,25]. A meta-analysis revealed a worldwide lifetime prevalence for NSSI of 17.2% among adolescents, ranging from 1.5% to 54.8% [26]. Moreover, repetitive NSSI was more strongly associated with severe mental health problems and suicidality [27]. Strikingly, suicide is a leading cause of adolescent death [28] and suicidal thoughts and behaviors (STBs) are elevated during adolescence [29,30]. In a German sample, the prevalence of suicidal ideation lay at 14.4% and 15.1% of girls and 10% of boys reported that they considered their lives as "not worth living" [31]. In general, data on the prevalence of suicide attempts among adolescents are equivocal, but probably range from 1.3% to 11.0%, with higher rates in girls [[32], [33], [34]]. In summary, early adolescence is a key period for the development of mental disorders. In particular, the increased occurrence of high-risk behavior and maladaptive emotion regulation strategies are reflected in very high rates of NSSI or suicidality.

Moreover, the restrictions during the COVID-19 pandemic have led to increased rates of mental health problems among adolescents in Germany, rising to 30% [35], thus adding another twist to the complex dynamics in adolescence. While the symptoms and rates of depression, eating disorders, suicidal behavior, and NSSI have increased across the world [[36], [37], [38], [39], [40]], to date, a nuanced investigation of a particularly young age cohort is lacking.

Given the described importance of early adolescence, this paper aims to provide deeper insight into the mental health status in early adolescence. In the current study, we therefore use self-report questionnaires answered by young adolescents to obtain prevalence rates to be used in future studies and clinical undertakings. Additionally, in view of the aforementioned gender differences reported in other studies worldwide, we also expected to find gender differences, insofar as girls would be generally more affected by mental health problems than boys would.

## 2 Methods

### 2.1 Study design

Data was drawn from the baseline assessment of a large prevention study and took place at seven German high schools [41]. Inclusion criteria were being in the 6^th^ or 7^th^ grade and informed consent of legal guardians and participating adolescents. The study was approved (127/19-me) and is conducted according to the guidelines of the Declaration of Helsinki and Good Clinical Practice (GCP).

### 2.2 Study population

In fall 2021, 877 adolescents aged 11-14 years participated in the baseline data collection. At the time of the baseline assessment, the number of pandemic-related restrictions was relatively low. While schools were open, the wearing of facemasks within all public spaces, including schools, was mandatory. In addition, vaccination for adolescents had become available and almost half of the German adult population had already been vaccinated, accompanied by a decrease in infection rates and deaths [42]. The seven high schools were located in cities (up to 130,000 residents) and rather rural areas (the smallest comprised 20,000 residents). All questionnaires were collected digitally in class on the participants’ own mobile telephones or school tablets and were selected based on acceptable to good psychometrics properties.

### 2.3 Measurements

Besides socio-demographic variables, we assessed socioeconomic status (SES) using the revised and shortened Family Affluence Scale (FAS III) [43]. The FAS III is a 6-item self-report scale measuring family wealth, which results in a low (index score 0-4), medium (index score 5-9), or high SES estimate (index score 10-14). The scale has shown moderate validity and a test-retest reliability of r = 0.90 [43], and can mainly be used to identify low- and high-income households [44].

### 2.4 General mental health problems

The *Strengths and Difficulties Questionnaire* (SDQ) is a widely used diagnostic instrument comprising 25 items (rated as “not true”, “somewhat true”, “certainly true”), which are allocated to five subscales (range 0–10). Additionally, a total difficulties score (range = 0-40; without the subscale prosocial behavior) is calculated on which a score of 17 indicates the cut-off for high and very high difficulties (www.youthinmind.com)*.* The five subscales of the SDQ are *emotional symptoms, conduct problems, hyperactivity/inattention, peer relationship problems,* and *prosocial behavior* (reverse-coded). Furthermore, an externalizing score (range 0 to 20; sum of conduct and hyperactivity scales) and an internalizing score (range 0 to 20; sum of emotional and peer problems scales) can be calculated. The SDQ has shown satisfactory to good reliability (Cronbach's alpha 0.73-0.89) and validity [35,45].The five subscales and an externalizing and internalizing score are depicted in Table 1.

**Table 1:** Sociodemographic and clinical characteristics for the total sample and for Girls, Boys and Non-Binary.

	Total	Girls	Boys	Non-Binary
Demographics				
N (%)	877	487 (56.0)	379 (43.2)	6 (0.8)
Age, mean (SD)	12.34 (0.65)	12.3 (0.64)	12.39 (0.66)	12.49 (0.82)
Range [min-max]	[11.08 - 14.92]	[11.17-14.17]	[11.08-14.92]	[11.25-13.17]
Participants born in Germany, n (%)	824 (94.0)	459 (94.3)	360 (95)	5 (83.3)
Father born in Germany, n (%)	735 (83.8)	408 (83.8)	323 (85.2)	4 (66.7)
Mother born in Germany, n (%)	720 (82.1)	400 (82.1)	317 (83.6)	3 (50.0)
Participants with				
siblings, n (%)	754 (86)	425 (87.3)	324 (85.5)	5 (83.3)
Chronic illness, n (%)	58 (6.6)	29 (6.0)	28 (7.4)	1 (16.7)
Mental illness, n (%)	16 (1.8)	10 (2.1)	5 (1.3)	1 (16.7)
in therapy^a^, n (%)	5 (0.6)	1 (0.2)	3 (0.8)	1 (16.7)
Strenghts and Difficulties				
SDQ total, mean (SD)	11.17 (5.36)	11.69 (5.56)	10.37 (4.91)	17.83 (6.49)
Range [min-max]	[0-30]	[1-30]	[0-26]	[11-26]
SDQ emotional, mean (SD)	2.98 (2.38)	3.52 (2.52)	2.25 (1.93)	5 (3.1)
Range [min-max]	[0-10]	[0-10]	[0-8]	[1-10]
SDQ conduct problems, mean (SD)	2.03 (1.52)	2.01 (1.52)	2.04 (1.50)	3.17 (2.56)
Range [min-max]	[0-8]	[0-8]	[0-8]	[1-7]
SDQ hyperactivity, mean (SD)	3.85 (2.17)	3.85 (2.14)	3.85 (2.22)	5.17 (1.83)
Range [min-max]	[0-10]	[0-10]	[0-10]	[3-8]
SDQ peer problems, mean (SD)	2.30 (1.62)	2.31 (1.57)	2.24 (1.67)	4.5 (1.05)
Range [min-max]	[0-9]	[0-9]	[0-9]	[3-6]
SDQ prosocial, mean (SD)	7.92 (1.81)	8.17 (1.70)	7.59 (1.89)	8.33 (1.86)
Range [min-max]	[0-10]	[2-10]	[0-10]	[6-10]
SDQ internalizing	5.28 (3.33)	5.83 (3.48)	4.49 (2.92)	9.5 (3.27)
Range [min-max]	[0-17]	[0-17]	[0-17]	[6-15]
SDQ externalizing	5.89 (3.17)	5.86 (3.17)	5.88 (3.15)	8.33 (3.53)
Range [min-max]	[0-17]	[0-17]	[0-17]	[4-13]
Depression				
PHQ-9, mean (SD)	4.75 (4.71)	5.36 (5.11)	3.87 (3.79)	10.6 (10.83)
Range [min-max]	[0-27]	[0-27]	[0-20]	[1-27]
mean (SD) above cut-off	15.62 (4.09)	15.89 (4.27)	14.42 (2.75)	21.5 (7.78)
Eating disorder (SEED)				
Anorexia, mean (SD) whole sample	1.42 (0.62)	1.51 (0.6)	1.31 (0.63)	1.56 (0.83)
Anorexia, mean (SD) above cut-off	2.36 (0.18)	2.37 (0.19)	2.32 (0.15)	2.5 (-)
n (%)^b^	106 (12.1)	68 (14.0)	37 (9.8)	1 (16.7)
Bulimia, mean (SD) whole sample	1.36 (0.41)	1.35 (0.44)	1.42 (0.29)	-
Bulimia, mean (SD) above cut-off	1.52 (0.39)	1.54 (0.44)	1.42 (0.29)	-
n (%)^c^	11 (1.3)	8 (1.6)	3 (0.8)	-
SES				
FAS III score, mean (SD)	10 (1.90)	10.02 (1.9)	9.98 (1.9)	9.5 (1.87)
Range [min-max]	[2-14]	[4-14]	[2-14]	[7-12]
FAS categorized, n (%)				
Low	2 (0.2)	1 (0.2)	1 (0.3)	0
Medium	343 (39.1)	185 (38.0)	155 (41.0)	3 (50.)
High	526 (60.0)	301 (61.8)	222 (58.7)	3 (50.0)

a According to the study protocol these participants were excluded from analysis.

b This number is based on a cut-off score > 2 equivalent to clinical relevant symptoms.

c This number is based on a cut-off score > 1 equivalent to mild clinical symptoms.

### 2.5 Mental disorders

#### 2.5.1 Depression

To assess depressive symptoms within the last two weeks, we used the nine-item *Patient Health Questionnaire* (PHQ-9; [46], which is a screening instrument based on the diagnostic criteria for major depression from the *Diagnostic and Statistical Manual of Mental Disorders, Fourth Edition* (DSM-IV) [47]. The nine items are rated on a 4-point Likert scale, with total scores higher than 10 indicating clinically relevant depressive symptoms (range = 0-27). The PHQ-9 is widely used among adults and has been validated for adolescents, showing good psychometric properties: sensitivity of 89.5% and specificity of 77.5% for detecting adolescents with major depression; Cronbach’s α = 0.83, test-retest reliability *rtt* = 0.87 [48].

#### 2.5.2 Eating disorders

The *Short Evaluation of Eating Disorders* (SEED) is a brief screening instrument for the main symptoms of AN and BN [49]. The instrument encompasses six items assessing body mass index (BMI) and the key eating disorder symptoms (fear of weight gain, distortion of body perception, over-concern with weight and shape, frequency of binge eating, frequency of compensatory behaviors). Item scores are converted to yield an individual severity index for AN and BN. To obtain prevalence estimates, we employed cut-off scores for AN (>2, clinically relevant symptoms) and BN (>1, mild clinical symptoms) regarding the an/bn_tsi scores in accordance with other studies [50,51]. The SEED is a widely used instrument and shows good psychometric properties [49].

#### 2.5.3 NSSI

NSSI was determined using a modified nine-item version of the *Deliberate Self-Harm Inventory* (DSHI-9) [52], which was adapted for adolescents [53] from the original 17-item version by Gratz. Respondents are first asked whether they have ever intentionally harmed themselves. If this question is answered in the affirmative, further questions on the frequency, method and severity are asked. The modified short version of the DSHI-9 shows moderate to good internal consistency (Cronbach’s α = 0.66 and 0.85) [54].

#### 2.5.4 Suicidal thoughts and behaviors (STBs)

Suicidal behaviors including suicidal thoughts, suicide plans, and suicide attempts were investigated using the *Paykel Suicide Scale* (PSS). The scale comprises five questions, which are answered on a 3-point Likert scale (“never”, “at an earlier time point”, “within the last two weeks”) [55]. If a student answered the first question *“Have you ever felt that your life is not worth living?”* in the affirmative (i.e., "at an earlier time point" or "within the last two weeks"), we considered the criterion of suicidality to be met; therefore, our estimate rate is based on suicidal thoughts. The PSS is a widely used questionnaire that shows good internal consistency (Cronbach's α of 0.85) [56].

#### 2.5.5 Statistical analyses

Analyses were performed with R and RStudio (version R.app GUI 1.74, RStudio 1.4.1106) using de-identified participant information. To calculate prevalence estimates, the cut-off values mentioned above were used. To investigate gender differences between prevalence estimates, t-tests were used for continuous data (Welch test in the case of unequal variances), Wilcoxon-Mann-Whitney tests were used for ordinal data and chi-squared tests were used for categorical data. In the case of significant differences, effect sizes were calculated using Cohen's d for independent means (small effect d = 0.2; medium d = 0.5, large d = 0.8), product-moment for z-values (small effect r = 0.1, medium = 0.3, large = 0.5) and Cramer’s V for chi-squared tests (small effect V = 0.1, medium effect V = 0.3, large effect V = 0.5) [57,58]. Note that due to the limited number of non-binary participants, they were not included in these tests. Effects were regarded as statistically significant at an alpha <.05.

## 3 Results

### 3.1 Socio-demographic characteristics

The sample comprised n = 877 adolescents with a mean age of 12.34 years (SD = 0.65). There were slightly more girls (55.5%) than boys (43.2%), and 0.7% of the adolescents identified as non-binary. The majority of participants (94.0%) and parents (83.0%) were born in Germany, and most of the participating adolescents had siblings (86.0%). The socioeconomic status in this sample was rather high, with 60% reporting a high socioeconomic status as measured by the FAS III and only 0.2% reporting a low socioeconomic status. A total of 6.6% of the participants were affected by a chronic illness, 1.8% stated that they had a psychiatric disorder, and 0.6% were in therapy. For further details on all socio-demographic information as well as means, SDs and ranges in the questionnaires used, see Table 1.

### 3.2 Prevalence estimates for mental disorders

#### 3.2.1 Mental health problems (SDQ)

Regarding mental health problems in general, the estimate was 12.5% within the last six months. Split by gender, the prevalence rate was 14.4% for girls, 9.2% for boys, and 50% for the non-binary group. There was a significant gender difference (*W* = 99404, *p* < .001) with a small effect size (*r* = 0.12), insofar as girls showed significantly more mental health problems than did boys. The mean scores on the different subscales of the SDQ differed slightly according to gender, with girls showing more emotional symptoms [3.52 (2.52) vs. 2.25 (1.93)] and more prosocial behavior [8.17 (1.70) vs. 7.92 (1.89)] compared to boys. There were no noteworthy gender differences on the rest of the subscales (see Table 1). Furthermore, a significant difference emerged on the internalizing problems scale [5.83 (3.48) vs. 4.49 (2.92)] with a small effect size (*W* = 99404, *p* < .001, *r* = 0.2), indicating more internalizing problems in girls than in boys. By contrast, no differences emerged on the externalizing problems scale.

#### 3.2.2 Depression (PHQ-9)

Regarding depression, we found an overall prevalence estimate of 11.5%, with 14.4% of girls and 9.2% of boys scoring above the clinically relevant cut-off. This difference was significant, with a small effect size (*t*(852.94) = 4.893, *p* < .001, *d* = 0.33). Girls had a higher mean score [5.36 (5.11)] than did boys [3.87 (3.79)], and non-binary participants had the highest mean score [10.6 (10.83)], indicating more depressive symptoms.

#### 3.2.3 Eating disorders (SEED)

The overall prevalence estimates for eating disorders were 12.1% for AN symptoms and 1.3% for BN symptoms. Half of the non-binary adolescents reported clinically relevant symptoms. More girls reported anorexic symptoms (14.0%) than did boys (9.8%). There was a significant difference between girls and boys regarding the anorexia severity index with a small effect size (*t*(831) = 4.82, *p* < .001, *d* = 0.34), but not for the bulimia severity index (*t*(14) = -0.261, *p* = .798). The girls had a slightly higher mean score than the boys [1.51 (0.6) vs. 1.31 (0.63)]. When considering only the participants scoring above the cut-off, the mean scores differed only slightly between girls and boys, regarding both AN symptoms [2.37 (0.19) vs. 2.32 (0.15)] and BN symptoms [1.54 (0.44) vs. 1.42(0.29)].

#### 3.2.4 NSSI (DSHI-9)

Overall, 10.8% of the sample reported having engaged in self-injurious behavior at least once in their lifetime. NSSI was reported by 12.3% of the girls, 9.0% of the boys and 16.7% of the non-binary participants. This difference was not significant for the whole sample (*χ*^*2*^(1) = 1.961, *p* = .161). However, when analyzing only those participants who had engaged in NSSI, a significant gender difference emerged (*χ*^*2*^(1) = 7.192, *p* = .007), with a medium effect size (Cramer's *V* = 0.28). Additionally, 5.59% of the sample reported engaging in repetitive NSSI. The main methods used were cutting (40%) and scratching (45.3%), followed by wound picking (36.8%), hitting oneself (32.6%), and burning (10.5%). As only one out of the six non-binary participants reported engaging in NSSI, the interpretation of these findings for the non-binary group is limited.

#### 3.2.5 STBs (PSS)

In total, 30.1% of the participants reported suicidal thoughts at any time point or within the last two weeks, 9.9% reported suicide plans, and 3.5% reported having attempted suicide in the past. When referring only to the last two weeks, these figures were as follows: 7.1% (n = 62) suicidal thoughts, 1.8% (n = 16) suicide plans, and 1.0% (n = 9) attempted suicide. Compared to boys, more girls reported suicidal thoughts (35.5% vs. 23.0%), had made suicide plans (11.7% vs. 7.4%), and had previously attempted suicide (3.7% vs. 1.6%). There was a significant gender difference for suicidal thoughts with a small effect size (*t*(853) = 4.477, *p* < .001, *d* = 0.31), for suicide plans (*t*(853) = 2.72, *p* = .007, *d* = 0.19), and for suicide attempts (*t*(853) = 2.678, *p* = .008, *d* = 0.19), suggesting more suicidal behavior overall among girls. It should be noted here that of the six adolescents who identified as non-binary, three stated suicide thoughts, two reported suicide plans, and one had previously attempted suicide.

An overview of the prevalence estimates can be found in Table 2. It can be observed that all six of the non-binary participants had higher scores on all questionnaires compared to girls or boys.

**Table 2:** Prevalence estimates in total and stratified by gender and grade.

		Total	Total sample (N = 871)			
			6th grade		7th grade	
			Girls	Boys	Girls	Boys
Mental health problems *(SDQ)*		105	33	16	37	19
	Prevalence^3^	12.49%	13.52%	9.04%	15.16%	9.22%
Depression *(PHQ-9)*	n	100	32	17	39	12
	Prevalence	11.48%	13.12%	9.61%	15.98%	5.83%
	Mean (*SD*)^1^	4.74 (4.70)	5.23 (4.98)	4.14 (3.91)	5.48 (5.24)	3.64 (3.67)
NSSI *(DSHI-9)*	n	94	25	23	35	11
	Prevalence	10.98%	10.25%	13.00%	14.34%	5.34%
Suicidal behavior *(PSS)^2^*	n	263	84	50	90	39
	Suicide thoughts	30.82%	34.43%	28.25%	36.89%	18.93%
	Suicide plans	10.08%				
	Suicide attempts	3.71%				
Eating disorder *(SEED)^4^*	n (AN)	106	44	22	24	15
	Anorectic symptoms	12.67%	5.26%	2.63%	2.87%	1.79%
	n (BN)	11	3	3	5	0
	Bulimic symptoms	1.31%	0.36%	0.36%	0.60%	-

^1^PHQ-9 range: 0-29. Values of 10 and higher indicate depression.

^2^ To determine this prevalence the answers "yes, within the last two weeks" and "yes, at a former time point", were added and divided by the total number.

^3^Based on cut-off score indicating high and very high scores.

NOTE: only two pupils being above the cut-off stated their gender as "divers"/non-binary, hence we did not include them in this table; f = 488, m = 383, d = 6.

^4^ The SEED cut-off scores are explained in the method section as well.

## 4 Discussion

With the current paper, we sought to provide an updated overview of the prevalence estimates of various mental health difficulties in early adolescence, which is known to be a critical period for the development and manifestation of mental health problems in general. To account for previously reported gender differences in adolescent mental health status, we also compared the obtained prevalence estimates between boys and girls.

In the whole sample, the six-month prevalence estimate for general mental health problems lay at 12.5%. Clinically relevant depressive symptoms within the last two weeks were reported by 11.5% of the participating adolescents. Although the prevalence estimate for general mental health problems found is lower than recently reported by another German longitudinal study [59], it is in line with a further German study [60] and slightly lower than pre-pandemic rates [61]. Accordingly, our results suggest no increased psychopathology in young German adolescents in temporal relation to the COVID-19 pandemic. Nevertheless, these results should be interpreted with caution, as given the observational nature of our data, we cannot draw any causal inferences about the participants' mental health status before the pandemic.

Furthermore, our results strongly suggest that girls are significantly more affected by mental health problems compared to boys. Regarding the subscales of the SDQ, we found significant differences between girls and boys on the internalizing scale. This finding is in line with previous literature, suggesting girls tend to be affected more by internalizing disorders such as depression or anxiety [62,63]. Surprisingly, we found no significant difference between boys and girls on the externalizing scale. This contrasts with the generally accepted assumption boys are more affected by externalizing symptoms than girls, which is also clinically reflected in the increased diagnostic frequency of AD(H)D in boys [10]. Potentially, this finding might be explained by the fact that AD(H)D is often underrated by parents and harder to diagnose in girls [64]. As such, the commonly observed difference in externalizing symptoms between boys and girls might be explained by a gap between self-perceived symptoms and third-party assessments (e.g., clinicians, parents). The current finding that girls and boys are equally affected by externalizing symptoms suggests that the ubiquitous conception of a higher frequency of externalizing symptoms in boys may be flawed, thus leading to an undersupply of treatment options and assistance for affected girls. Future research utilizing multi-informant assessment strategies is needed to further investigate gender-specific differences in the diagnostic frequency of externalizing symptoms.

The prevalence estimate of 11.5% of adolescents screening positive for depressive symptoms is in line with findings from before the pandemic [65] and with a recent longitudinal German study [59]. Nevertheless, other studies have revealed a mixed body of evidence, with some reporting lower rates of depressive symptoms in adolescents during the pandemic [60] and others reporting an increase in depressive symptoms [66]. Thus, while our findings suggest that the pandemic has not led to an increase in rates of depressive symptoms in this age group, the estimates reported here are not fully comparable to previous studies due to the lower mean age of our sample and the use of different assessment procedures, and different assessment time points. Further, due to the volatile nature of the ongoing COVID-19 pandemic, it could be that at the time of data assessment, the participants in the current study had fewer depressive symptoms due to relatively mild restrictions. Resilience research has shown that children who were already struggling with risk and developmental harm (poverty, racism, neglect, food insecurity, violence, or chaos in the home) prior to the pandemic have become even more isolated and stressed due to the pandemic. Therefore, high resilience relative to the overall population may be another factor explaining the findings in our sample [67]. More generally, the effects of COVID-19 on prevalence estimates remain largely unknown, calling the comparability of (inter-)national prevalence estimates over the last two years into question. However, our results suggest a comparably high rate of depressive symptoms overall and that girls are significantly more affected. More longitudinal research, especially in young adolescents, is needed to verify the prevalence of self-reported depressive symptoms found in the present study and investigate the effects of the COVID-19 pandemic in greater depth.

For eating disorder symptoms, the prevalence estimates in the present study was 12.1% for anorexic symptoms and 1.3% for bulimic symptoms. Girls were significantly more affected by anorexic symptoms, while this was not the case for bulimic symptoms. These estimates are in line with a multicenter study conducted in 2019, which found similarly high rates, especially for girls [21]. While the prevalence estimate of 12.1% for AN seems surprisingly high, it corresponds to a previous study which distinguished eating disorders into a partial and full syndrome [20]. Specifically, the latter study reported a similar proportion of participants fulfilling criteria for only a partial syndrome to the present study. Accordingly, our results may reflect an overestimation of full syndrome AN, while nevertheless raising concerns given the high proportion of participants meeting the criteria for a partial syndrome. Moreover, the high prevalence estimate in the present study may be explained by the fact that some eating disorder habits resemble the eating habits commonly observed in adolescence, such as being on a diet, feeling too fat, and counting calories. In addition, our participants self-reported their height and weight. This may have led to a bias in the prevalence estimate for AN, as the calculated BMI is essential for the anorexia severity index. Lastly, the recently observed worldwide increase in patients with an eating disorder since the onset of the COVID-19 pandemic [68,69] may also be an explanation. With regard to bulimic symptoms, the prevalence of 1.3% is in line with other studies, which suggested that BN symptoms emerge at later time point in adolescence [20,70]. Further research is needed, particularly in the context of validation and standardization of the SEED in adolescence, as there are no general cut-offs or norms available for this age group. Given the complex nature of eating disorders in general and of AN in particular, future research should rely on clinical interviews to assess the prevalence rates of eating disorders.

For NSSI, a dysfunctional coping behavior often associated with mental health problems, we found a 12-month prevalence estimate of 10.8%. This finding is lower compared to a European study from 2014, which found a lifetime prevalence of 17.1% to 38.6% across various countries [52]. Another meta-analysis found estimates ranging from 6% to 26% in a comparable age range worldwide [71]. Therefore, our results indicate a moderate occurrence of NSSI in early adolescence. A meta-analysis [72] showed that women were significantly more likely to report a history of NSSI than men, with no significant association between age and effect size. In terms of methods of NSSI, women were more likely than men to use some NSSI methods (here: cutting and scratching), but there was no significant difference for other methods (here: burning, wound picking, hitting). These results are consistent with the present findings. However, research suggests that more girls engage in NSSI, with associations between NSSI and mental health difficulties [73]. Another possible explanation for the occurrence of NSSI in adolescence and the partially found gender differences lies in females’ increased vulnerability to internalizing disorders. A cluster analysis confirmed that especially girls who engage in cutting often show higher scores on the SDQ difficulties scale and on the internalizing scale, while this association was not found in boys [54]. Accordingly, girls who have more internalizing problems appear to have a higher vulnerability to NSSI. More generally, the assessment of NSSI is complicated by the lack of consistent definition of self-harm and consequently the fact that there is no standard questionnaire to measure NSSI, rendering it difficult to draw international comparisons. Given these limitations, we consider our findings to be preliminary and a starting point for future epidemiological investigations.

30.1% of adolescents reported suicidal ideation, 9.9% had planned suicide and 3.5% had attempted suicide. These estimates are broadly consistent with the literature, with a 2008 study reporting 12-month prevalence estimates of 15.0% to 29.0% for suicidal ideation and 12.6% to 19.0% for suicide plans [74]. Interestingly, the present study found a very high prevalence of suicidal ideation. A recent article pointed out that in the US, suicide rates have increased by 300% in the last 10 years [75]. When testing for gender differences, we found significant differences regarding suicidal thoughts, plans and attempts, with girls being more affected than boys. There is still a large gap in research on the mechanisms of self-harm and STB/suicide in early adolescence. However, again, there is some evidence of increased prevalences in females, which are associated with hormonal changes during puberty [76], and it is suggested that this should be addressed using a more individual and social approach. Moreover, the well-known gender paradox of suicide (higher prevalence of suicidal behavior in women yet men are more likely to die by suicide [28]) is in line with our findings of more girls being affected than boys.

Other notable aspects of our data, besides capturing pandemic-related effects, include the high SES of the sample. Considering that low SES tends to be a risk factor for increased stress [77,78], it may also have been assumed that prevalence estimates for mental disorders should be rather low in a sample with a rather high SES such as in the current study [79]. As reported, this is only partly the case, as at least partially high symptom rates were reported, especially for girls and for non-binary individuals. These findings are therefore contrary to expectation, and underline that SES cannot explain the effects found in the present sample. Potentially, the impact of the COVID-19 pandemic may have moderated the results, reflecting the vulnerability in this particular age group.

### 4.1 Strengths and limitations

When interpreting the present results, some limitations should be taken into account. First, the present data come from a cross-sectional observation with limited sample size and a distinct region. Generalization of our findings to other populations is therefore limited. Second, our estimates rely entirely on self-report and may thus be affected by social desirability and demand characteristics. For example, participants might have been reluctant to report their honest feelings in response to items assessing mental health problems, which are still subject to public stigmatization. Nevertheless, to the best of our knowledge, the present study is the first to assess prevalence estimates of mental health problems and mental disorder symptoms in adolescents within the selected age range of 11–14 years. Which is known to represent an important time for the onset of mental disorders in general [1]. Despite limited generalizability, our findings therefore provide important insights into a particularly vulnerable population. Moreover, the COVID-19 pandemic itself poses another limitation. The different data collection periods of individual studies assessing the impact of the pandemic make it difficult to compare results in general, as exposure to COVID-19 fluctuates worldwide, and changes in the course of a pandemic influence mental health on an individual basis. Nonetheless, our data contribute to some degree gaining an understanding of the dynamics surrounding COVID-19 in a selective sample assessed 1.5 years after the outbreak.

### 4.2 Conclusion

Our findings underline the profound problem of mental health difficulties and mental disorders in youth, which should finally be given the necessary public attention in order to secure and maintain the mental health of our young people. A societal rethink on both a social and political level is required, not only to protect our health care system in the long term, but also to strengthen the younger and following generations to cope with the demands of daily life. According to our findings, it is essential that researchers and clinicians pay greater attention to gender differences regarding the onset and course of mental disorders in early adolescence. Moreover, it is important that adolescents are strengthened at an early stage to enable effective and efficient prevention at the right time. Given the vulnerability to mental disorders during adolescence, there is an urgent need for intervention and preventive approaches. Such interventions could be routinely delivered in schools. Moreover, adolescents’ parents or guardians should be sensitized to potential issues faced by their children and should be given guidance in providing the necessary help to prevent mental health difficulties. Preventive interventions can be subdivided into selective or universal approaches. Selective programs aim to reduce risk in those who benefit most and therefore reach a small proportion of the population, while universal approaches aim to reduce the risk of a more general population [1]. According to our results, we should therefore ask ourselves what is the most appropriate and effective way to strengthen adolescent mental health in early adolescence in order to increase resilience and protect against mental illness in the long term.

## Author affiliation

CS helped design and coordinate the study, participated in its coordination, wrote the manuscript, and did the statistical analysis; JG participated in the study coordination and helped draft the manuscript; NK helped draft the manuscript and did the statistical analysis; AB conceived the study and participated in its design and coordination and helped draft the manuscript. All authors read and approved the final manuscript.

## Funding

Kaufmännische Krankenkasse (KKH). The funder does not influence the collection, analysis or interpretation of data or the writing of this or any following article on DUDE.

KKH (Kaufmännische Krankenkasse); Referat Prävention und Selbsthilfe; Mr. Tobias Bansen; Karl-Wiechert-Allee 61; 30625 Hannover; Germany.

## Ethics

The Ethics Review Committee of the University of Würzburg approved the trial (127/19-me). The Ministry of Education and Cultural Affairs has also approved the trial (IV.7-BO5106/200/12). Written informed consent from the participants and their parents was mandatory for participation.

## Declaration of competing interest

The authors declare that they have no known competing financial interests or personal relationships that could have appeared to influence the work reported in this paper. This publication was supported by the Open Access Publication Fund of the University of Wuerzburg.
